# Current stage of the intensive care unit structure in Argentina: results from the *Sociedad Argentina de Terapia Intensiva* self-assessment survey of intensive care units

**DOI:** 10.5935/0103-507X.20220021-en

**Published:** 2022

**Authors:** Ramiro Gilardino, Antonio Gallesio, María Pilar Arias-López, Nancy Boada, Verónica Mandich, Judith Sagardia, Maria Elena Ratto, Ariel Fernández

**Affiliations:** 1 Comité de Gestión, Calidad y Datos, Sociedad Argentina de Terapia Intensiva - Buenos Aires, Argentina.

**Keywords:** Intensive care units/ organization & administration, Intensive care units/classification, Critical care/ organization & administration, Structure of services, Health facilities

## Abstract

**Objective:**

To describe and compare the structure of Argentinean intensive care units that completed the “self-assessment survey of intensive care units” developed by the *Sociedad Argentina de Terapia Intensiva*.

**Methods:**

An observational crosssectional study was conducted using an online voluntary survey through the *Sociedad Argentina de Terapia Intensiva* member database and other social media postings. Answers received between December 2018 and July 2020 were analyzed. Descriptive statistics and nonparametric tests were used.

**Results:**

A total of 392 surveys were received, and 244 were considered for the analysis. Seventy-seven percent (187/244) belonged to adult intensive care units, and 23% (57/244) belonged to pediatric intensive care units. The overall completion rate was 76%. The sample included 2,567 ICU beds (adult: 1,981; pediatric: 586). We observed a clear concentration of intensive care units in the Central and Buenos Aires regions of Argentina. The median number of beds was 10 (interquartile range 7 - 15).

The median numbers of multiparameter monitors, mechanical ventilators, and pulse oximeters were 1 per bed with no regional or intensive care unit type differences (adult versus pediatric). Although our sample showed that the pediatric intensive care units had a higher mechanical ventilation/bed ratio than the adult intensive care units, this finding was not linearly correlated.

**Conclusion:**

Argentina has a notable concentration of critical care beds and better structural complexity in the Buenos Aires and Centro regions for both adult and pediatric intensive care units. In addition, a lack of accurate data reported from the intensive care unit structure and resources was observed. Further improvement opportunities are required to allocate intensive care unit resources at the institutional and regional levels.

## INTRODUCTION

Over the last 15 years, there has been an increase in the demand for critical care services worldwide.^(1,2)^ Demographic changes, aging populations, the rise in non-communicable diseases, and mass casualties contribute to ever increasing needs for intensive care units (ICUs).^(3)^ By contrast, there is an unresolved shortfall of ICUs, which causes conflicts in situations in which the demand could exceed the health care capability.^(4)^

Measuring the physical, technological, and human resources in the ICU can help us to understand and quantify this burdensome problem, to establish policies to reduce this shortage. Categorizing ICUs allows benchmarking among facilities, establishing quality improvement programs, and could also support the regionalization of critical care delivery.^(5-7)^ Although several publications address ICU resources throughout surveys, little is known in low-to-middle income countries or where critical care medicine is still in development.^(7-11)^

The *Sociedad Argentina de Terapia Intensiva* (SATI) developed the first Argentine ICU Categorization Guidelines in 1986. At that time, in conjunction with the National Social Security Institute, the society drafted the first-ever “Standards for the structural, organizational and human resources profile of an ICU” to be used for reimbursement and later on in accreditation initiatives and in the National Quality Assurance program of the Ministry of Health (MoH).^(12)^

From 2010 - 2014, a joint task force integrated by representatives of the SATI’s Management, Quality and Scores Committee and Pediatric Chapter, as well as the Critical Care Committee from the *Sociedad Argentina de Pediatria* (SAP), reviewed and updated the former standards by considering the technology and scientific advances in the field and the growing demands for pediatric and adult critical care resources.

According to these Argentinean ICU Guidelines, ICUs are categorized based on their ability to solve specific critical illnesses and their technological resources. A level 1 ICU (ICU-1) provides the highest multidisciplinary care to critically ill or injured patients, while in level 2 (ICU-2), general critical care is available around the clock. Health care facilities that usually provide care for low-risk diseases and do not have an ICU must have a resuscitation unit (Resus) to provide stabilization until further transport to an ICU can be arranged.^(13)^

In December 2018, SATI implemented the “self-assessment survey of intensive care units” (“self-assessment survey”), a voluntary survey to assess the degree of adherence to the ICU Categorization Guidelines and to compare and understand how critical care is being delivered across the country.

We aim to describe the current stage of the critical care structure in Argentina by analyzing the technology and resources from the ICUs.

## METHODS

We performed an observational, cross-sectional, retrospective study by employing a “self-assessment survey” received between December 2018 and July 2020.

The “self-assessment survey” consists of 145 items divided into general characteristics of the ICU and the Institution; staffing and human resources; structure (ICU, patient area, and working areas); supporting specialties and infrastructure (i.e., availability of computerized tomography (CT), magnetic resonance imaging (MRI), renal replacement therapies, etc.), as well as process of care and quality indicators. This work provides an estimate of the degree of compliance for facilities to be qualified as an ICU-1, ICU-2, Resus, or intermediate ICU. A table 1S (Supplementary material) describing survey components can be found in the supplementary material. The survey has been available on SATI’s webpage (https://www.hardineros.com.ar/liveform2/index.php/461171) since December 2018.

SATI’s Board of Directors approved the “survey” used to collect data. No further institutional review board was required for this type of study. No humans or animals were involved in the study.

### Study population and data collection

With this voluntary survey, we encouraged the participation of Argentinean ICUs through email invitations to ICU medical directors at SATI’s member database, including a link at SATI’s webpage, and promotion through social media network posts.

Prior to the analysis, the self-assessment surveys were anonymized and blinded to the investigators. Personal data and other sensitive information were stored securely in compliance with local data protection regulations.

If a center submitted more than 1 survey from the same ICU, we included the most recent submission for the analysis, and previous responses were disregarded. Data from multiple ICUs within the same center were analyzed separately. The survey did not collect patient information.

### Selection of the variables

For the purposes of this study, we decided to analyze the following variables:

### Intensive care unit characteristic variables

- **Payer:** Public payer (i.e., public hospital, funded by national, provincial, or municipal MoH); private payer (private hospitals, social security or private insurance institutions)
- **Health care provider level of care:** Tertiary (advanced level of care facilities); secondary (intermediate level of care facilities)
- **Type of ICU:** Adult and pediatric ICUs included the medical-surgical and specialized units (i.e., neurotrauma, infectious diseases, transplant, and cardiovascular care units). Although we analyzed subgroups within the specialized units, they were not reported separately
- **University affiliation**

### Structure variables

- Number of institutional beds
- Number of critical care beds in the institution (i.e., overall number in the ICU, intermediate ICU), Resus, step-down and post-anesthesia care beds from the participating institution)
- Number of adult or pediatric ICU beds (from the participating unit)
- Availability and number of the following devices: multiparameter monitoring (MM); invasive blood pressure (IBP); pulse oximeter (SO_2_); mechanical ventilators (MV); and non-invasive ventilation (NIV)
- Device/ICU bed ratios were determined to better characterize resource availability

The National Institute of Statistics and Census (INDEC - *Instituto Nacional de Estadísticas y Censos*) database (https://www.indec.gob.ar/ftp/cuadros/publicaciones/anuario_estadistico_2017.pdf) was employed to determine the overall institutional and critical care beds as well as the beds/100,000 inhabitants and MV/100,000 inhabitants. Intensive care units were classified into six regions across the country: Buenos Aires (including Buenos Aires Metropolitan City and Buenos Aires province); Cuyo; Northeast; Northwest; Central; and Patagonia.

### Data analysis

Surveys containing at least four indicators from the structure were analyzed; to avoid bias and confusion, surveys without data from MM, IBP and MV were disqualified.

Continuous variables are presented as the median and interquartile range (IQR), while categorical variables are presented as frequencies and percentages.

### Statistical analysis

The Kruskal-Wallis one-way analysis of variance and Mann-Whitney U test were employed to compare continuous variables, and the chi-square test was employed for categorical variables. Odds ratios (ORs) and 95% confidence intervals (95%CIs) were employed to express the strengths of associations between the type of unit (adult or pediatric ICU) and the type of payer.

Linear and weighted least squares regression was used to adjust for potential confounders (payer and country region) of the association between MV/bed ratio and the pediatric ICU, taking into account that the model did not meet the assumptions for linear regression, especially homoscedasticity.

A p value < 0.05 was considered statistically significant. The Stata V14.0 package was used to run the statistical analysis.

## RESULTS

After the removal of duplicates and incomplete surveys, 244 were analyzed (“the sample”). An overall 76% completion rate was observed across our sample (as defined by the completion of questions related to the general facility, ICU staffing, supporting services, ICU area and ICU structure - Table 1S - Supplementary material).

**Figure 1:**
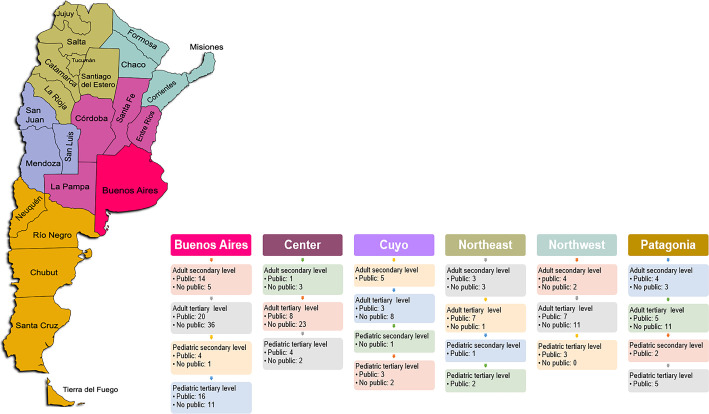
Number of participating intensive care units divided per Argentina region.

Source: modified from: Instituto Nacional de Estadística y Censos (INDEC). Anuário Estadístico de la República Argentina 2017. Ciudad Autónoma de Buenos Aires. Buenos Ayres: INDEC; 2018. [cited 2022 Apr 27]. Available from: https://www.indec.gob.ar/ftp/cuadros/publicaciones/anuario_estadistico_2017.pdf

Intensive care units in the public (121/244; 49.5%) and private (123/244; 50.41%) settings were represented similarly, and 44% of the units were university affiliated. The level of care of participating ICUs is described in figure 1.

The median number of hospital beds in the institutions that completed the survey was 105 (94 - 125); this response represented a total of 34,740 institutional beds and 87 beds/100,000 inhabitants. The total number of critical care beds was 4,639 and represented 12% of the total beds. The medians were 13 critical care beds/institutions and 12 critical care beds/100,000 inhabitants. A detailed analysis across country regions is presented in table 1.

**Table 1:** Overall hospital and critical care resources in our sample

Region of Argentina	Population (M)	Demography Area Density (Km^2^)		Participating units	Total beds	Hospital beds IQR Median (25 - 75)		Beds/100,000 inhabitants	Total CC beds	Median CC beds/hospital	CC beds/ hospital IQR (25 - 75)	Critical care setting Ratio CC/ hospital beds	CC beds/100,000 inhabitants	MV/100,000 inhabitants
Buenos Aires	18,515	307,571	60	107	17,793	165	(65 - 280)	96	2,413	16	(8 - 37)	10%	13	5
Cuyo	2,852	315,226	9	22	3,388	93	(42 - 220)	119	366	12	(6 - 28)	13%	13	5
Center	8,058	377,109	21	41	4,130	100	(46 - 128)	51	716	11	(8 - 24)	11%	9	4
Northeast	3,680	289,699	13	17	2,207	110	(36 - 180)	60	280	14	(8 - 17)	13%	8	3
Northwest	4,911	559,864	9	27	4,113	130	(82 - 202)	84	648	20	(11 - 34)	15%	13	4
Patagonia	2,100	1,752,888	1	30	894	81	(53 - 145)	43	407	12	(8 - 19)	15%	19	10
Total	40,117	3,602 M	11	244	34,740	105	(94 - 125)	81	4,639	13		12%	12	6

IQR - interquartile range; CC - critical care; MV - mechanical ventilators.

Notes: Buenos Aires region includes the city of Buenos Aires (metropolitan area) and Buenos Aires Province. For regional composition, refer to figure 1.

Source: modified from: Instituto Nacional de Estadística y Censos (INDEC). Anuário Estadístico de la República Argentina 2017. Ciudad Autónoma de Buenos Aires, INDEC; 2018. Available from: https://www.indec.gob.ar/ftp/cuadros/publicaciones/anuario_estadistico_2017.pdf

### Structure analysis

The sample represented 77% of adult ICUs (187/244) and 23% of pediatric ICUs (57/244), accounting for 2567 ICU beds, of which 586 were pediatric ICU beds. We provide a descriptive analysis of the six variables of interest.

The overall beds per participating ICU were adult ICU 10 (7 - 16) and pediatric ICU 8 (6 - 14). The Buenos Aires, Center, and Northeast regions showed a higher number of beds (13 [7 - 20]; 9 [7 - 12,5] and 10 [8 - 14], respectively). Additionally, the Buenos Aires region showed a higher median of specialized ICU beds, 19 (6,25 - 19). In contrast, all the regions showed uniform pediatric ICU bed distribution.

The overall ratio of MM/bed was 1 in both the adult and pediatric ICUs. In a cross-comparison between regions, the Northeast and Northwest registered ratios < 1 for both adult (0.85 - 0.88) and pediatric ICUs (0.75 - 0.94), this difference was not significant for the total sample.

The survey reported that 62% of the overall ICUs had access to an IBP measurement (either as an MM rack or a separate machine); however, there were differences across the three types of units. While pediatric ICUs had a ratio of 1 IBP/bed, there were regional variations, in the Northeast and Patagonia, which showed ratios < 0.8 In the case of the adult ICUs, the difference was more profound, with an overall ratio of 0.45, especially in the Cuyo and Center regions, where this ratio was below 0.35.

The ratio of SO_2_/bed was 1 for both the adult ICU and pediatric ICU, without regional variations and differences across payers.

Our sample reported an MV/bed ratio of 1 in both the adult and pediatric ICUs, 1 (0.67 - 1.08) *versus* 1 (0.87 - 1.17). Adult ICUs in the Central, Northeast, and Northwest regions showed ratios below 0.75.

The aim of question on the NIV was to understand how many continuous-flow devices to perform NIV are present in the ICUs. Overall, 44% of ICUs could deliver continuousflow NIV with a median device of 3 in adult ICUs and 5 in pediatric ICUs. These values represented a ratio of 0.34 in the adult ICUs *versus* 0.55 in the pediatric ICUs.

Tables 2 and 3 provide the geographical and type of payer comparisons for each resource across the adult and pediatric ICUs.

**Table 2:** Geographical distribution and structure comparison according to intensive care unit type

	Total (244)	Adult ICU (187)	Pediatric ICU (57)	p value	OR (95%CI)
Geographical distribution					
Buenos Aires	107 (43.85)	75 (40.11)	32 (56.14)		
Center Cuyo	41 (16.80) 22 (9.02)	35 (18.72) 16 (8.56)	6 (10.53) 6 (10.53)	0.23	
Northeast	17 (6.97)	14 (7.49)	3 (5.26)		
Northwest	27 (11.07)	24 (12.83)	3 (5.26)		
Patagonia	30 (12.29)	23 (12.30)	7 (12.28)		
Payer					
Public payer^*^	121 (49.59)	81 (43.32)	40 (70.18)	< 0.001	3.08 (1.57 - 6.21)
University hospital	108 (44.26)	73 (39.04)	35 (61.4)	0.003	2.49 (1.30 - 4.80)
Tertiary center	188 (77.05)	140 (74.87)	48 (84.21)	0.14	
Structure†					
Beds number	10 (7 - 15)	10 (7 - 16)	8 (6 - 14)	0.15	
MM/bed ratio	1 (1 - 1.1)	1 (1 - 1.10)	1 (1 - 1.17)	0.056	
MV/bed ratio	1 (0.66 - 1.1)	1 (0.67 - 1.11)	1 (0.87 - 1.17)	0.0044	
SO_2_/bed ratio	1 (1 - 1.07)	1 (1 - 1)	1 (1 - 1.2)	0.05	

ICU - intensive care unit; OR - odds ratio; 95%CI - 95% confidence interval; MM - multiparameter monitor; MV - mechanical ventilator; SO_2_ - pulse oximeter.

* Public hospitals or facilities run by the local, state or national government; † Mann-Whitney U test. The results are expressed as the median/(interquartile range).

**Table 3:** Structure comparison according to geographical region and type of payer

	Beds^*^	p value	MM/bed ratio^*^	p value	MV/bed ratio^*^	p value	SO_2_/bed ratio^*^	p value
Geographical distribution								
Buenos Aires (107) Center (41) Cuya (22)	11 (7 - 18) 9 (7 - 12.5) 6 (5 - 16)	0.24	1 (1 - 1.14) 1 (1 - 1.08) 1 (1 - 1.17)	0.19	(0.79 - 1.08) 1(0.6 - 1.17) 1.06 (0.83 - 1.2)	0,27	1 (1 - 1.12) 1 (1 - 1) 1 (1 - 1.23)	0.42
Northeast (17)	10 (8 - 14)		1 (0.63 - 1)		1 (0.86 - 1.25)		1 (0.85 - 1)	
Northwest (27)	10 (6.5 - 15.5)		1 (1 - 1.12)		0.7 (0.41 - 1.16)		1 (0.8 - 1.08)	
Patagonia (30)	9 (6 - 12)		1 (1 - 1.11)		1 (0.66 - 1.04)		1 (0.67 - 1.04)	
Payer†								
Public‡ (121)	8 (6 - 14)	0.028	1 (1 - 1.17)	0.018	1 (0.9 - 1.17)	< 0.001	1 (1 - 1.15)	0.84
Private§ (123)	10 (7.5 - 16.5)		1 (1 - 1.02)		0.8 (0.62 - 1)		1 (1 - 1)	

MM - multiparameter monitor; MV - mechanical ventilator; SO_2_ - pulse oximeter.

* Kruskal-Wallis one-way analysis of variance; † Mann-Whitney U test; ‡ public hospitals or facilities run by the local, state or national government; § private institutions or facilities run by social security, private insurers or for-profit corporations. Results expressed as the median (interquartile range).

We employed linear regression to adjust for potential confounders, such as the payer and country region association with the MV/bed ratio with pediatric ICUs. The type of payer showed a significant association with the MV/Bed ratio (p > 0.001) and region of the country (p = 0.04) in the univariate analysis.

In addition, we found that our model did not meet the homoscedasticity assumptions; hence, weighted least squares were applied, indicating that the pediatric ICU showed higher MV/bed ratios, independent of the country region.

It is important to note that even when comparing the variables of interest, none of the ICUs participating in the survey met the requirement stated in the National Guidelines for ICU categorization.

## DISCUSSION

The main results can be summarized as follows: a greater concentration of critical care beds and better structural complexity in the Buenos Aires and Centro regions for both adult and pediatric ICUs, including specialized critical care services, with a median of 9 and 13 beds/100,000 inhabitants, respectively. Intensive care units from public payers have better technological resources; in addition, pediatric ICUs are better equipped than adult ICUs, especially those from public payers.

A total of 4,830 critical care beds were reported, and 2,567 were analyzed, representing 20.9% of Argentinean ICU beds. Larger urban centers usually concentrate most of the health structure, including specialized hospitals, trauma centers, and ICU-1.^(1,6,8,11,14)^ Considering that the ICU bed number is calculated based on the number of inhabitants and that it should not be less than 15/100,000,^(4,14)^ our sample shows that the number of beds might be insufficient in a situation of increased demand, such as a pandemic.

Data collected from the Integrated Health Information System (SISA - *Sistema Integrado de Información Sanitaria*) before the coronavirus disease 2019 (COVID-19) pandemic accounted for 8,527 critical care beds, which have been expanded to 12,547 in the last year to meet the COVID-19-related ICU demand.^(15)^ We created a cut-off for the surveys on 01/04/2020 and found that 36 surveys were completed after this date (10% of the total), which is not representative of generalizing an increase in structural resources during the COVID-19 surge.

When analyzing technological resources, in some regions, the ICUs of public payers appear to be better equipped than those of private payers. Although private ICUs reported a higher median number of ICU beds, we observed an inverse relationship in MV/bed and MM/ bed ratios; in contrast, the SO_2_/bed ratio was not affected.

Moreover, pediatric ICUs tend to comply with MV requirements since all regions reported a statistically significant ratio above the national standards (MV availability for at least 70% of their beds); however, this difference disappears when linear regression adjusts it to public payers.

Notably, 70% of the pediatric ICUs were from large urban centers, and 60% were university affiliated. In the Buenos Aires and Center areas, both public and private health care facilities require better technological structures to cope with severe pediatric diseases. Outside large urban centers, as described before, public hospitals are the first to provide care to critically ill or injured children until they can obtain a referral to a tertiary center, which is why they need to have the necessary facilities (at least a pediatric ICU-2).^(16)^

Pediatric ICU categorization guidelines were developed jointly by SATI and SAP, which represent the entire pediatric critical care community in Argentina.^(13,17)^ The mentioned factors could impact adherence to the recommendations and support why our sample indicated that pediatric ICUs had better equipment ratios.

Although there might be a selection bias due to the voluntary participation of the ICUs and the characteristics of this survey, the fragmentation of the Argentine health care system, in which the public, social security, and private sectors coexist and overlap,^(18)^ could have contributed to this finding.

In Argentina, the public sector provides coverage to 34.1% of the population, and the remainder is a mix of social security, the private sector, or both.^(19)^ Regarding private health care providers, variability in their structure is observed, in which we can look from Resus to an ICU-1 with the ability to resolve the most critical illnesses, such as organ transplantation or cardiovascular surgery.

Nonetheless, accessibility to health care, whether public or private, is conditioned by providers and payers (public and private). Outside large urban centers in Argentina, public health care providers must sometimes resolve critically ill and acute pathology due to a lack of resources in the private sector. This concept supports the fact that ICUs from public payers have better structures and are inserted into providers with the capabilities to provide 24/7 availability of diagnostic services (CT, MRI) or blood banks on site. However, this relatively good structure does not necessarily imply that this structure is sufficient to meet the demand. Furthermore, the exclusion of staffing data from our analysis could affect the results and require consideration before making conclusions. By using examples of other areas in the economy, we explore the role of public-private partnerships in which the public sector could support the provision of care and the private sector could enhance the technological resources to improve critical care delivery.

To date, Argentina has not published accurate data about its human and technological ICU resources. This lack of information could be explained by how licensing and accreditation processes in health care facilities are established. First, the MoH has been developing health care facility categorization guidelines that include the ICU level according to the services provided and the level of the health care facility. Although this information may be available in the SISA, in-depth surveys to understand the state of the ICUs in the country are needed. The second point is that institutional accreditation and the categorization of services are not mandatory in this country. Until that happens, surveys such as ours are considered voluntary.

The ICU categorization guidelines were agreed upon by the MoH as requirements to achieve progressively and without any punitive measures for those who did not meet. Although the ICUs had lengthy physical and technological resources, they had not fulfilled all the requirements established by the MoH to qualify for an ICU-1 or ICU2 category. We sought to standardize the resources of ICUs across these three subsectors. Moreover, to become a national policy, these guidelines should be agreed upon by the Mercosur Health Council, in which all the members discuss and standardize the technical, human, and physical resources in the ICUs of the state members.

Nonetheless, ICU categorization must consider the staffing (physicians, nurses, respiratory therapists and other allied professionals) in both number and capabilities. The policies, procedures, protocols, and processes of care contribute to improving the quality of critical care that is delivered, improving patient and family satisfaction, and encouraging their involvement in the care process; hence, this information must be available.^(7,20-24)^

In that context, the “self-assessment survey” was developed to provide a trustworthy and reliable tool with which Argentinean ICUs could assess their structure, staffing, and processes of care and implement improvement measures accordingly. Although we did not analyze the staffing and processes of care, it was demonstrated that Latin America, like any other low-to-middle income region, has great opportunities for improvement in the mentioned areas.^(16,20,25-27)^ This survey did not include specific outcome measurements or ask about patient outcomes. However, a higher percentage of the respondents remarked that they measure patient outcomes and quality indicators, with most of them participating in the Quality Benchmarking SATI-Q Program sponsored by the SATI. The SATI-Q encompasses the voluntary participation of public and private ICUs with different levels of complexity.

The participating units collect data about the patients admitted to the ICU, their outcomes, and a set of quality indicators in a standardized format, and annual reports for adult and pediatric ICUs are available on the program website (https://www.satiq.net.ar/informes).

### Strengths and limitations

This study represents the greater real-world data survey collection of ICU information in Argentina, because we had representation from both public and private payers and adult and children’s facilities from every region of the country. It highlights the considerable variations in the structure across all regions in the country. However, several limitations are important to note. First, because participation was voluntary, this study might not reflect the “real” current stage of ICUs across Argentina; although we tried to gather responses from all the regions, most of them came from large urban centers. Due to selection bias, it is not possible to generalize the results. The survey was lengthy, demanding time to convene and fill in with the requirements; hence, many incomplete responses might affect the response validity.

## CONCLUSION

The analysis of the structure and technology through the self-assessment surveys made clear the need for having accurate and updated data about the intensive care unit capabilities and resources at the country level. This is reliable information for supporting decision-making and implementing policies about the role of intensive care units across the entire health care ecosystem, the future of providing critical care outside the limits of the intensive care unit, and what would happen if the demand exceeded the available services. Classifying health facilities according to their capabilities and resources, regionalizing critical care provision, and employing telemedicine or remote-intensive care units could help mitigate the never-ending shortage of critical care resources.
