# Supplementary material for: Current stage of the intensive care unit structure in Argentina: results from the Sociedad Argentina de Terapia Intensiva self-assessment survey of intensive care units
Source: Rev Bras Ter Intensiva. 2022 Apr-Jun;34(2):237–46. doi: 10.5935/0103-507X.20220021-en (PMC9354106; doi:10.5935/0103-507X.20220021-en)
Supplement: Supplementary file 1 [file rbti-34-02-0237-suppl01.pdf]

Current stage of the intensive care units structure in Argentina: results from the *Sociedad Argentina de Terapia Intensiva* self-assessment survey of intensive care units

Cenário atual da estrutura das unidades de terapia intensiva na Argentina: resultados do inquérito de autoavaliação de unidades de terapia intensiva da Sociedad Argentina de Terapia Intensiva

Ramiro Gilardino[1], Antonio Gallesio[1], María Pilar Arias-López[1], Nancy Boada[1], Verónica Mandich[1], Judith Sagardia[1], Maria Elena Ratto[1], Ariel Fernández[1]

Table 1S - Variables included in the intensive care unit categorization guidelines

Variables		Pediatric ICU	Adult ICU	Level of unit			
				ICU-1	ICU-2	Resus	Intermediate ICU
Supporting medical specialties and other services	Anesthesiology with pediatric expertise, 24-hour on-duty	X		M			
	Anesthesiology with pediatric expertise, 24-hour on-call	X			M		M
	Pediatric surgery, 24 hour on-duty or available within 1 hour, if there's a general surgeon 24-hour on-duty	X		M	M		M
	Anesthesiology, 24-hour on-duty		X	M	M	R	M
	Anesthesiology, 24-hour on-call		X			M	
	General surgery, 24-hour on-duty		X	M	M	R	M
	Neurosurgery, 24 hour on-duty or available within 1 hour.	X	X	M	M	R	M
	Orthopedics 24 hour on-duty; availability to consult with pediatric orthopedics	X		M			
	Orthopedics 24-hour on-duty		X	M	M	R	M
	Orthopedics 24-hour on-call		X		M	M	M
	Pediatric cardiology, 24-hour on-call	X		M	M		M
	Digestive/respiratory endoscopy, 24-hour on-call	X	X	M	M	R	M
	Possibility of consultation with specialties not considered in the previous items	X	X	M	M	R	M
	Hemotherapy and blood bank, 24-hour availability (on duty)	X	X	M	M	R	M
	Hemotherapy and blood bank, 24-hour availability (on-call)	X	X			M	
	Radiology availability 24-hour (XR technician on-duty)	X	X	M	M	R	M
	Portable X-ray machine available for the ICU	X	X	M	M		M
	Computerized tomography on-site, 24-hour available	X	X	M	R	R	R
	Computerized tomography outside the facility, 24-hour accessible.	X	X		M	M	M
	Laboratory services available 24/7	X	X	M	M	M	M
	Magnetic resonance imaging on-site or 24-hour avaiable.	X	X	M	M	R	M
	Ultrasound available within 60 minutes	X	X	M	M	R	M
	Interventional Rx on-call 24/7	X	X	M	M	R	M
ICU area	Not less than 4 - 6 beds or 1000 days/patients/year	X		M			
	Not less than 8 beds/unit		X	M	M	R	M
	Not less than 4 beds	X	X		M	M	M
	Intermediate ICU: not less than 8 beds and not exceed in 2 the overall ICU beds		X				M
	Minimum total area: number of beds x 7m2 x 2 (open areas)	X		M	M		
	Minumum surface: 9 - 12m2 (for ICU open areas)		X	M	M	M	
	Working area/patient area ratio, minimum 50 - 50%	X	X	M	M	R	R
	Working area/patient area, minimum 30 - 70%	X	x			M	M
	Isolation areas: minimum total surface 9m2 (pediatric ICU) 12m2 (ICU) per bed	X	X	M	M	R	R
	At least 1 bed and 9 m2	X	X			M	M

Continue...

...continuation

	Variables	Pediatric ICU	Adult ICU	Level of unit			
				ICU-1	ICU-2	Resus	Intermediate ICU
Patient zone	On-site stock of medicines	X	X	M	M	M	M
	Handwashing facilities, minimum 1 per 4 beds; alcohol-based hand sanitizer per bed	X	X	M	M	M	M
	Handwashing facilities, 1 per 2 beds; Alcohol-based hand sanitizer per bed		X	M	M	M	M
	Windows	X	X	R	R	R	R
	Walls (special treatment)	X	X	M	M	M	M
	Sanitary plinth	X	X	M	M	M	M
	Oxygen outlets, 2/bed	X	X	M	M	M	M
	Central vacuum outlets, 1/bed (preferably 2)	X	X	M	M	M	M
	Compressed air outlets, minimum 1/bed	X	X	M	M	M	M
	Oxygen, vacuum, and compressed air: at least 1/bed		X			M	
	General lighting on the ceiling approximately 100W, night light. Intense light for procedures	X	X	M	M	M	M
	Doors opening that allows the passage of stretchers and equipment	X	X	M	M	M	M
	Direct patient visualization	X	X	M	M		M
	Isolation room	X	X	M	M		R
	Alarm system (coding alarm) per bed	X	X	M	M	R	M
	Electrical system: 10 outlets/bed. Plug for portable x-ray machine	X	X	M	M		R
	Electrical system. 4 outlets/bed. Plug for portable x-ray machine	X	X			M	M
	An air-conditioned system, conveniently filtered.	X	X	M	M	M	M
Equipment	Multiparametric monitor, 1/bed (ECG, invasive/non-invasive blood pressure, temperature, pulse oximetry)	X	X	M	M	R	R
	Multiparametric monitor, 1/bed (ECG, noninvasive blood pressure, pulse oximetry)	X	X			M	M
	Capnograph monitoring every 4 patients on mechanical ventilation	X	X	M	M		
	Capnograph monitoring device, 1 per unit	X	X		M		
	Mechanical ventilators available for 100% of beds (micro processed 80%)		X	M	R		
	Mechanical ventilators available for 70% of beds (micro processed 80%)	X		M	M		
	Mechanical ventilators available for 75% of beds (micro processed 80%)		X		M	R	
	Mechanical ventilator for the unit (at least 1)	X	X			M	M
	High-frequency oscillatory ventilator, at least 1	X		M	M		
	Emergency kart, as standard, 1 each sector	X	X	M	M	M	M
	Precision infusion pumps, 4/bed (0.1 - 999mL/h)	X	X	M	M	R	R
	Precision infusion pumps, 3/bed (0.1 - 999mL/h)	X	X		M		
	Precision infusion pumps, 2/bed (0.1 - 999mL/h)	X	X			M	M
	Enteral feeding pumps, 1 every 2 beds	X	X	M	R		R
	Enteral feeding pumps, 1/ bed	X	X	R			
	Portable electrocardiograph, 1 per unit	X	X	M	M		
	Portable electrocardiograph available	X	X			M	M
	External pacing (wire and generator), 1 every 3 ICU beds	X	X	M			
	External pacing (wire and generator), 1 in the ICU	X	X	M	R		
	Resuscitation bag-valve-mask with reservoir device, 1/ bed	X	X	M	M	M	M
	Vascular access devices from 4 F to 7 F (2 per size)	X	X	M	M		M
	Equipment for intra-osseous access, 1 per unit	X	X	M	M	M	M
	Minute volume computer or dock	X	X	R	R		
	Equipment for venous dissection, 1 per unit	X	X	M	M		M
	Chest drainage equipment	X	X	M	M	M	M
	Punction equipment (lumbar, abdominal)	X	X	M	M	M	M
	Peritoneal dialysis equipment	X	X	M	M		M
	Renal-replacement therapy available 24/7	X	X	M	R		
	Portable ultrasound available	X	X	M	M		M
	Color Doppler echocardiography available	X	X	M	M		R
	Transcranial Doppler	X	X	R	R		
	Equipment for patient's temperature management	X	X	M	M		
	Portable X-ray machine available for the unit	X	X	M	M		M
	Intracranial pressure monitoring device available	X	X	M	M		
	Transport bed/stretcher with portable multi-parameter monitor and oxygen	X	X	M	M	M	M
	Decubitus ulcers prevention devices	X	X	M	M		R

Continue...

...continuation

Variables		Pediatric ICU	Adult ICU	Level of unit			
				ICU-1	ICU-2	Resus	Intermediate ICU
Supporting zone	Nursing station	X	X	M	M		M
	Nursing and cleaning staff lounge	X	X	M	M		M
	Equipment storage	X	X	M	M		M
	Clean linen storage	X	X	M	M		M
	Area for stocking of medicines and disposable material	X	X	M	M	M	M
	Dirthy utilityl	X	X	M	M		M
	Drug refrigerator	X	X	M	M	M	M
	Locked compartment for narcotics and concentrated electrolytes	X	X	M	M	M	M
	Independent refrigerator for feeding formulas	X	X	M	M		M
	Soiled workroom	X	X	M	M		M
	Washing and preparation area for surgical material	X	X	M	M		M
	Administrative assistants area	X	X	M	M		M
	Family reception area	X	X	R	R		M
	Medical staff lounge	X	X	M	M		M
	Offices for the medical and nursing director (independent)	X	X	M	M		M
	Physician on-call rooms	X	X	M	M		M
	Staff toilet	X	X	M	M		M
	Patient waste disposals	X	X	M	M		M
	Fire protection measures, evacuation plan	X	X	M	M		M
	Internet access, availability of online medical libraries	X	X	M	M		M
Documentation	Diagnosis, date of admission/discharge, demographic data	X	X	M	M	M	M
	Standardized mortality ratio	X	X	M	M		
	State at discharge	X	X	M	M		M
	Healthcare-related adquired Infections acquired in the pediatric ICU	X	X	M	M		
	Adverse events (unplanned extubating, falls from bed, pressure sores)	X	X	M	M		M
	At least 1 process indicator	X	X	M	M		
Indicators	Diagnostic and treatment guidelines (common diseases in the ICU)	X	X	M	M		M
	Nursing care protocols	X	X	M	M		M
	Quality improvement and patient safety program	X	X	M	M		R
	Informed consent policy	X	X	M	M	M	M
	Infection control program	X	X	M	M		M
	Invasive procedures protocol	X	X	M	M	M	M
	CPR and advanced life support care standards	X	X	M	M	M	M

ICU - intensive care unit; ECG - electrocardiogram; CPR - cardiopulmonary resuscitation; M - mandatory; R - recommended.
